# Supplementary material for: Repression of Irs2 by let‐7 miRNAs is essential for homeostasis of the telencephalic neuroepithelium
Source: EMBO J. 2020 Sep 28;39(21):e105479. doi: 10.15252/embj.2020105479 (PMC7604626; doi:10.15252/embj.2020105479)
Supplement: Supplementary file 1 — Expanded View Figures PDF [file EMBJ-39-e105479-s001.pdf]

Expanded View Figures

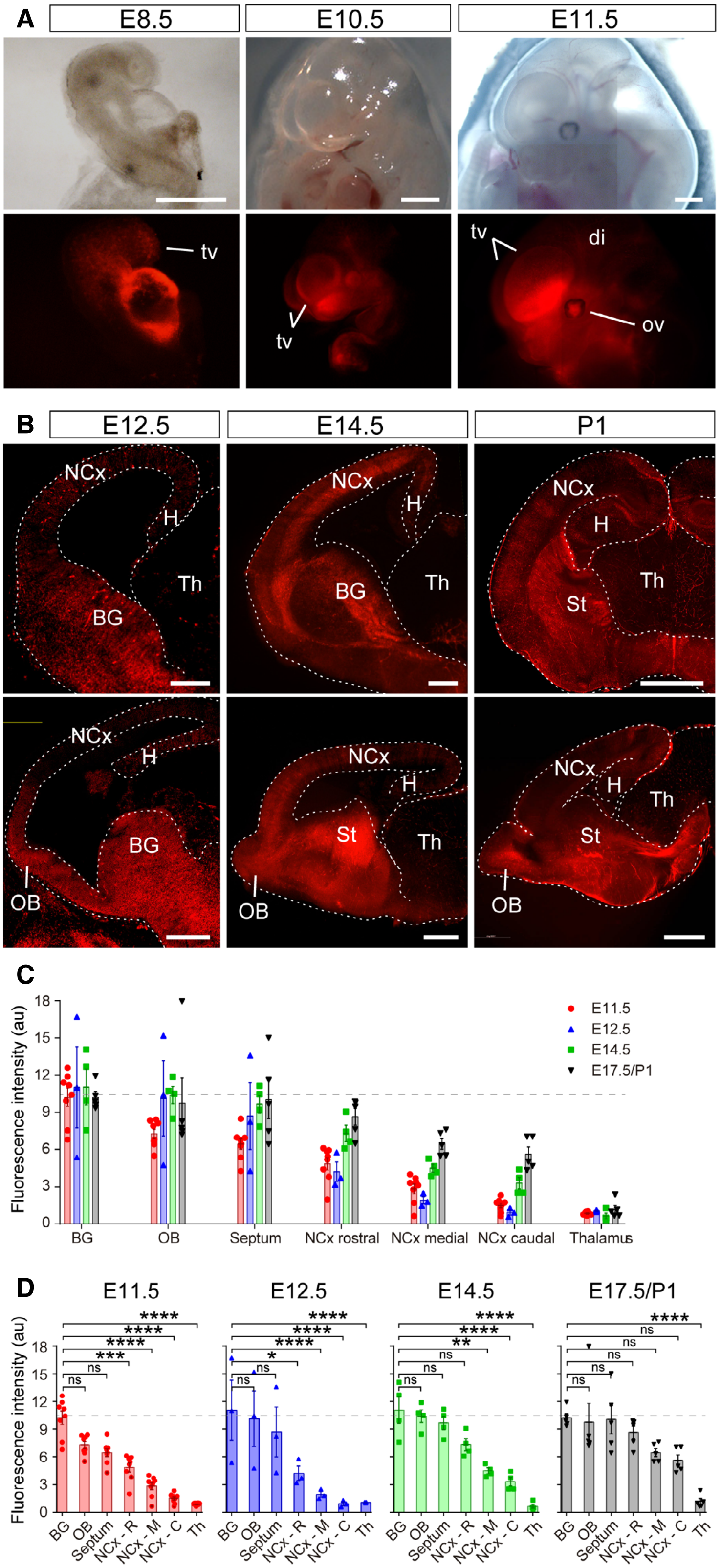

Figure EV1. Early recombination in the dorsal telencephalon of Rx-Cre mice.

A, B Bright field (top row) and TdTomato expression (red) under the *Rx3* promoter as seen in whole embryos (A) and brain sections (B) at the embryonic ages indicated. Sections in (B) are in coronal (upper panels) and sagittal plane (bottom panels). BG, basal ganglia; di, diencephalon; H, hippocampus; NCx, neocortex; OB, olfactory bulb; ov, optic vesicle; St, striatum; Th, thalamus; tv, telencephalic vesicle. Scale bars, 500 μm (A), 300 μm (B).

C, D Quantification of Tomato fluorescence intensity upon Rx-Cre recombination in the forebrain areas and at the embryonic stages indicated. BG, basal ganglia; NCx, neocortex; OB, olfactory bulb. Dashed line indicates average level of Tomato fluorescence in Basal Ganglia. Comparison across telencephalic areas at each stage is shown in (D). Data in histograms are mean ± SEM, symbols indicate individual values; n = 3–8 replicates per age. ANOVA followed by Tukey's test; ns, not significant; *$P < 0.05$, **$P < 0.01$, ***$P < 0.001$, ****$P < 0.0001$. See Table EV1 for the full set of statistical results.

© 2020 The Authors

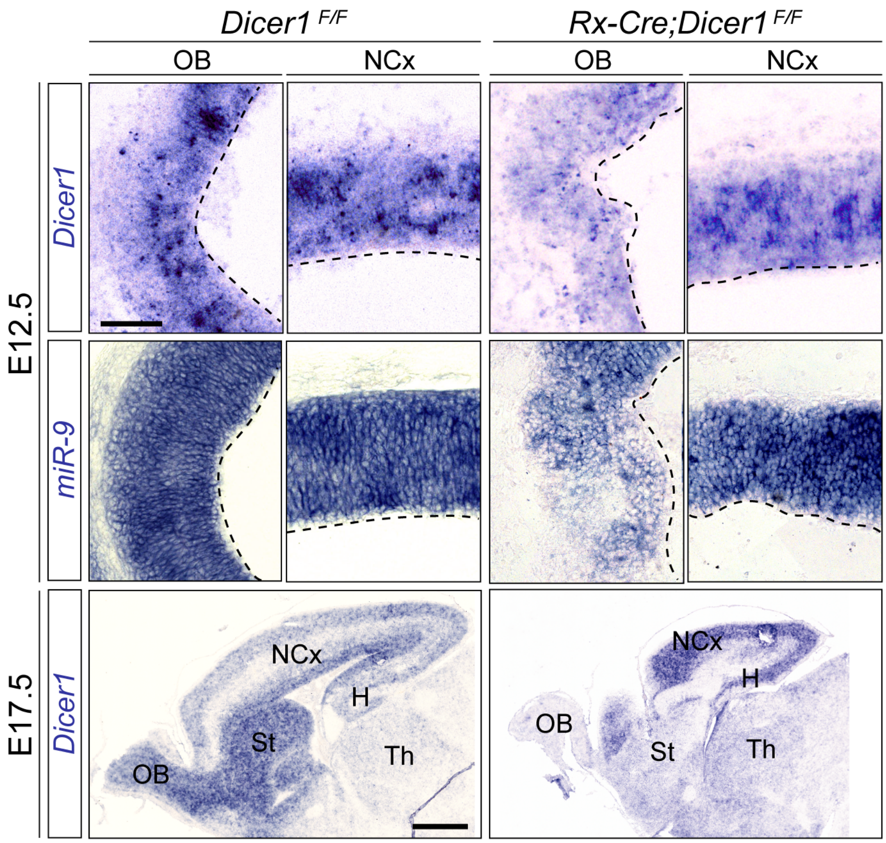

Figure EV2. Early loss of telencephalic *Dicer* and *miRNAs* in *Rx-Cre;Dicer*$^{F/F}$ mutant mouse embryos.

Sagittal sections of control (*Dicer*$^{F/F}$) and *Rx-Dicer* (*Rx-Cre;Dicer*$^{F/F}$) mutant embryos at E12.5 and E17.5 showing the expression of *Dicer1* mRNA and *miR-9* in the olfactory bulb (OB) and neocortex (NCx). Dashes line is apical surface. Levels of *Dicer* and *miR9* expression were dramatically reduced in the OB and subpallium of mutants. St, striatum; H, hippocampus; Th, thalamus. Scale bar, 100 µm (E12.5, all images at the same scale), 1 mm (E17.5, all images at the same scale).

© 2020 The Authors

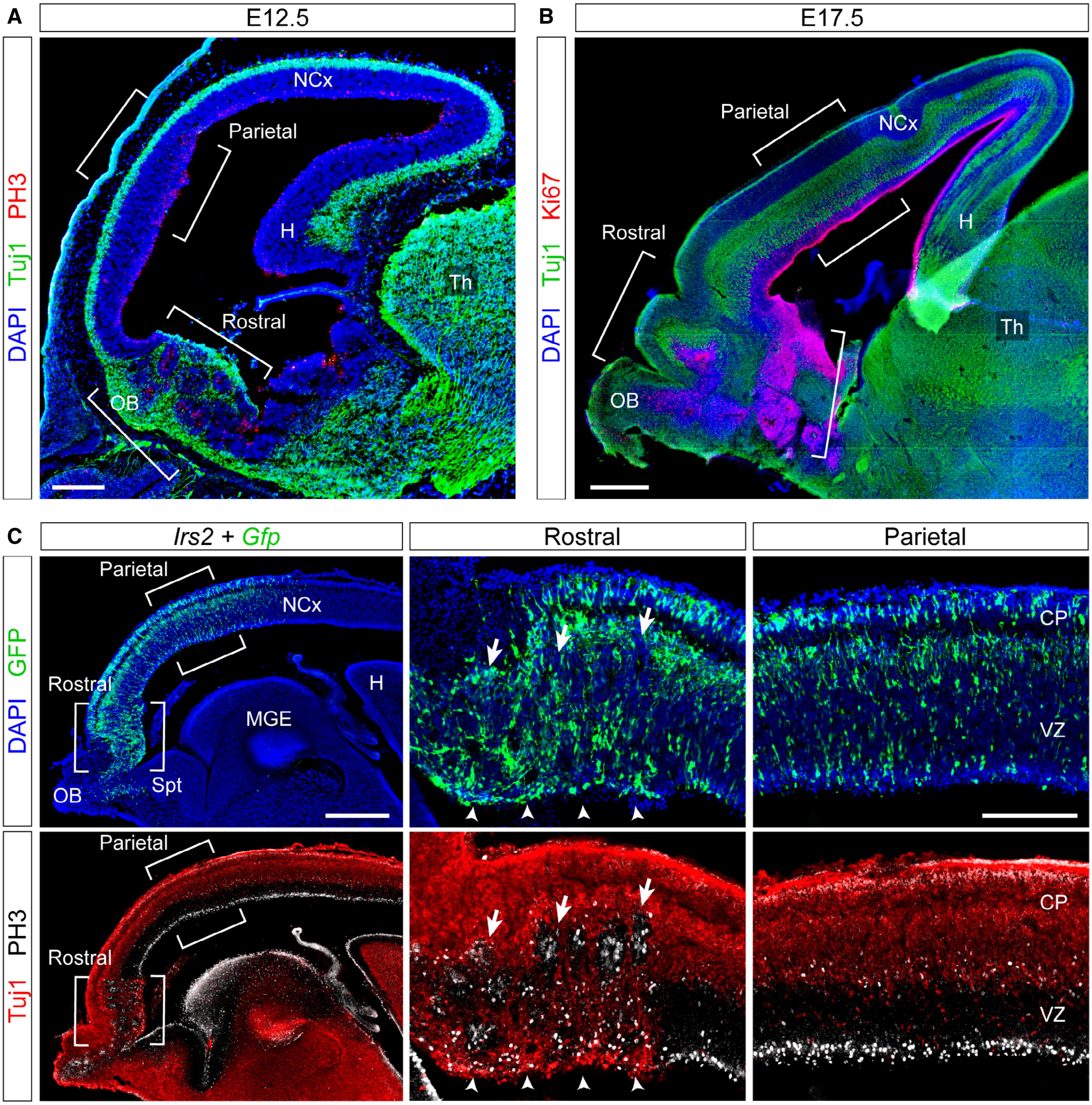

Figure EV3. Regionalized formation of rosettes in Dicer mutants and Irs2 overexpression.

A, B Sagittal sections through the telencephalon of *Rx-Dicer* mutant embryos at the indicated ages stained for the indicated markers, showing the formation of proliferative rosettes selectively in the rostral but not the parietal (or caudal) regions, as indicated with brackets.

C Sagittal section through the telencephalon of an E14.5 wild-type embryo electroporated with *Irs2* + *Gfp* encoding plasmids across the entire rostral and parietal regions of the neocortex, stained with the indicated markers. Details of each region are shown on the right. Arrows indicate proliferative rosettes, and arrowheads indicate ectopic Tuj1^{+} neurons in the apical border of the VZ. The electroporated parietal neocortex (NCx) remained unaffected, with perfect layering of neurons, and apical and basal mitoses.

Data information: H, hippocampus; MGE, medial ganglionic eminence; OB, olfactory bulb; Spt, septum; Th, thalamus. Scale bars, 150 μm (A), 500 μm (B, C), 100 μm (C, details).

© 2020 The Authors

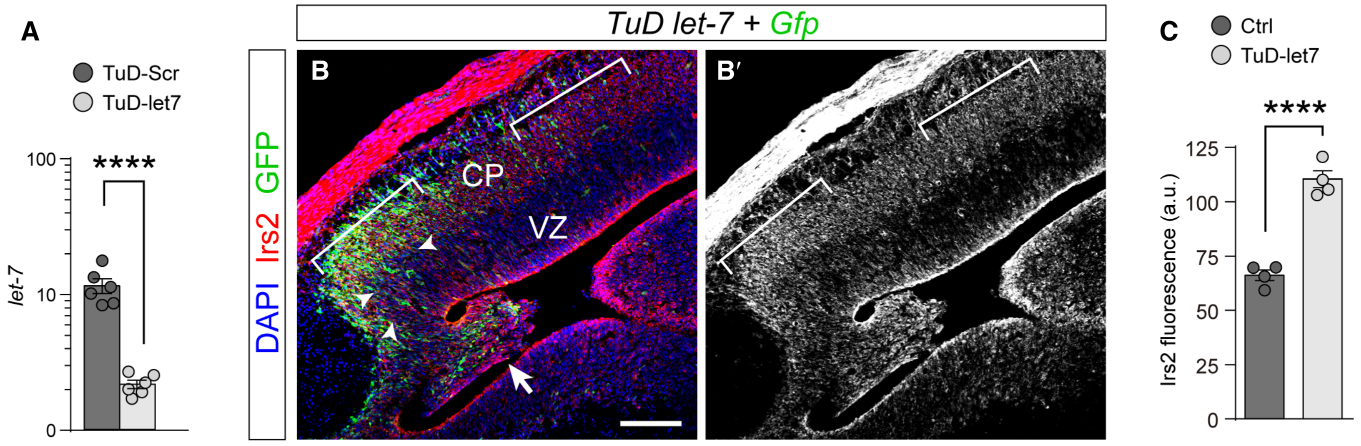

Figure EV4. Loss of *let-7* in the rostral cerebral cortex leads to increased Irs2.

A qPCR quantification of endogenous levels of mature *let-7* in HEK cells upon transfection with *TUD-Scr* or *TUD-let7* encoding plasmids. Mean ± SEM and individual data points are shown on a logarithmic scale; $n = 6$ replicates per group; *t*-test, ****$P < 0.0001$.

B, C Sagittal section of a wild-type embryo electroporated with *TuD let-7 + Gfp* plasmids at E12.5 and analyzed at E14.5 for the immunodetection of Irs2 protein (red, white), and quantification (C). Brackets indicate the electroporated area of neocortex (green) and the adjacent control area compared. Mean ± SEM and individual data points are shown; $n = 4$ replicates per group; *t*-test, ****$P < 0.0001$. Levels of Irs2 almost doubled upon expression of *TuD-let7*. Rosettes are indicated by arrowheads, and the typical ventricular neuronal ectopia caused by the loss of *let-7* is indicated by an arrow.

Data information: Scale bar, 100 µm (B).

© 2020 The Authors

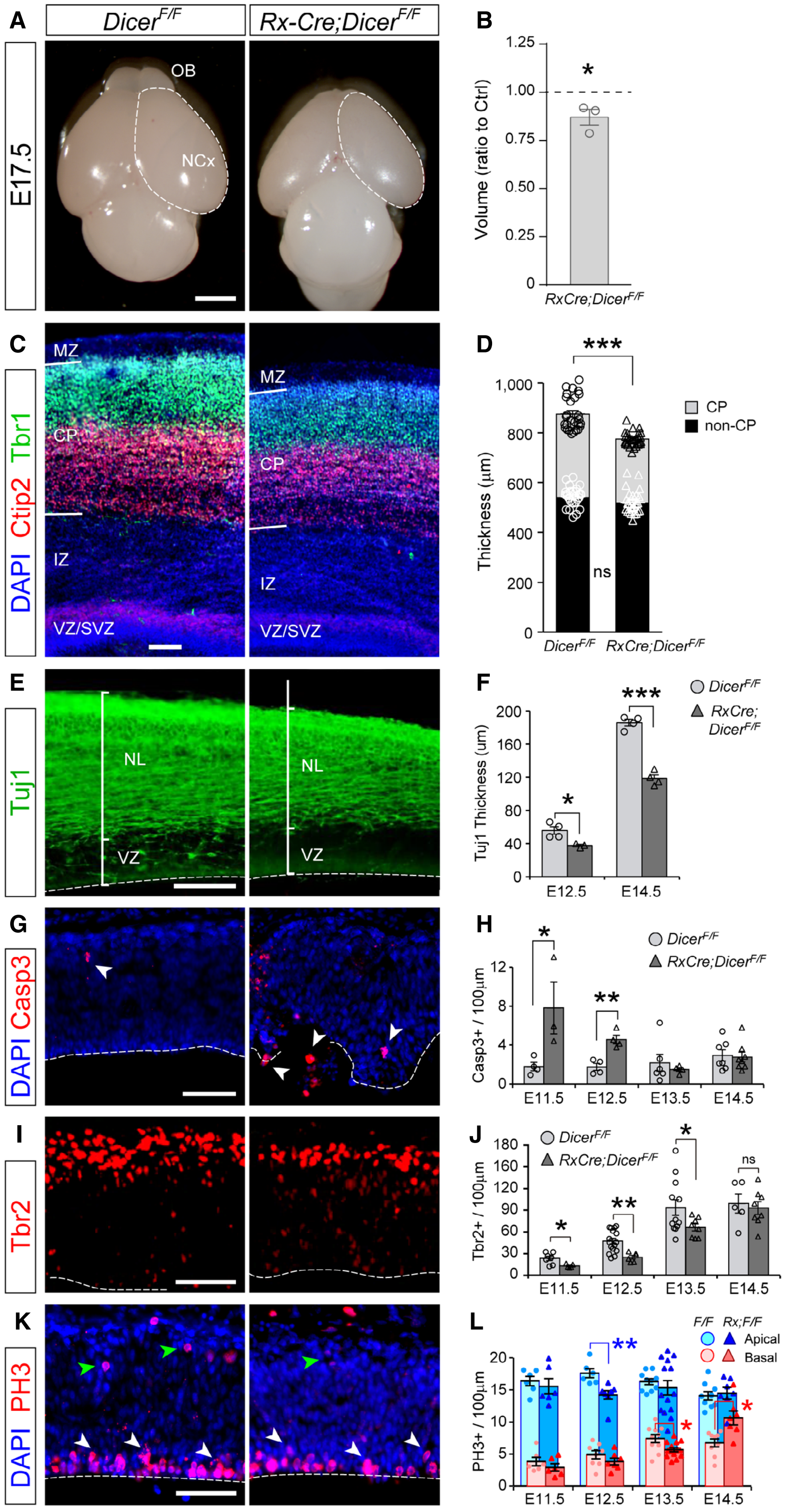

Figure EV5. Mild neocortical defects in *Rx-Dicer* mutant embryos.

- A External view of the brains of control (*Dicer*$^{F/F}$) and *Rx-Dicer* mutant littermates (*Rx-Cre;Dicer*$^{F/F}$) at E17.5, showing the reduction in cerebral cortex (NCx) size and apparent loss of olfactory bulb (OB) in mutants. Dashed lines indicate border of NCx.
- B Quantification of telencephalic volume (values relative to control littermates). *N* = 3 replicates.
- C, D Location and thickness of cortical plate (CP), superficial (Tbr1^{+}), and deep (Ctip2^{+}) cortical layers, in control and *Rx-Dicer* mutants, and quantifications. *N* = 27 replicates per group. Black symbols = CP, white symbols = non-CP. MZ, marginal zone; IZ, intermediate zone.
- E, F Thickness of the layer containing neurons (Tuj1^{+}) in the E14.5 cerebral cortex of control and *Rx-Dicer* mutant embryos, and quantifications. Dashed line indicates apical surface. NL, neuronal layer. *N* = 4 replicates per group.
- G–L Distribution and quantification of apoptotic cells (Casp3^{+}), Tbr2^{+} cells, and apical and basal mitoses (PH3^{+}) in the cerebral cortex of E12.5 control and *Rx-Dicer* mutant embryos. Dashed line indicates apical surface; white arrowheads indicate apical mitoses, and green arrowheads indicate basal mitoses. *N* = 4–14 replicates per group.

Data information: Histograms indicate mean ± SEM; symbols in plots indicate values for individual embryos; *t*-test; ns, not significant, **P* < 0.05, ***P* < 0.01, ****P* < 0.001. Scale bars: 1 mm (A), 100 µm (C–K).

© 2020 The Authors
